# Magnetic resonance imaging-guided ultrasound ablation for prostate cancer – A contemporary review of performance

**DOI:** 10.3389/fonc.2022.1069518

**Published:** 2023-01-04

**Authors:** Mostafa Alabousi, Sangeet Ghai

**Affiliations:** Toronto Joint Department of Medical Imaging, University Health Network-Mt Sinai Hospital-Women’s College Hospital, University of Toronto, Toronto, ON, Canada

**Keywords:** prostate cancer, MRI, Prostate MRI, MRI-guided intervention, Focal Therapy, HIFU, ultrasound, prostate ablation

## Abstract

Prostate cancer (PCa) is one of the most common malignancies in men, but patient outcomes are varied depending on extent of disease. Radical, whole-gland therapies, such as prostatectomy or radiotherapy, are definitive treatments for PCa, but they are associated with significant morbidity, including erectile dysfunction and urinary incontinence. Focal therapies for PCa, whereby the part of gland harboring disease is selectively treated, spares the normal surrounding structures, and minimizes the morbidity associated with whole gland treatment. The use of magnetic resonance imaging (MRI) guidance provides advantages over ultrasound guidance, such as better localization and targeting of clinically significant PCa (csPCa), as well as MRI thermometry which optimizes tissue ablation temperatures. This review will discuss two MRI-guided high-intensity focused ultrasound (HIFU) techniques – transrectal MR-guided focused ultrasound (MRgFUS) and TULSA (transurethral ultrasound ablation) ablation for localized PCa. Overall, recent major trials for MRgFUS and TULSA have shown promising oncological and functional results in the treatment of low- to intermediate-risk PCa. Recent Phase II MRgFUS trials have shown better oncologic outcomes than the published results for focal ultrasound guided HIFU and may justify the additional costs associated with MRI guidance. While initial studies on TULSA have focused on subtotal gland ablation, recent trials assessing oncological outcomes for focal treatment of angular sectors have shown promise.

## Introduction

Prostate cancer (PCa) is one of the most commonly diagnosed malignancies in men worldwide (1). Patient outcomes are varied depending on the grade and extent, ranging from localized disease to regional spread and distant metastasis (2). Consequently, risk stratification, including the use of tumor stage (TNM staging), tumor grade (Gleason score), and prostate specific antigen (PSA) levels, has played an important role in guiding management of men with PCa (3, 4). Whole-gland therapies for localized PCa, such as radical prostatectomy or pelvic radiotherapy with or without systematic therapy, have served as a definitive treatment approach, but this method is not without its challenges (5). Notably, radical prostatectomy and radiotherapy can result in significant erectile dysfunction in more than half of treated men, while many experience long-term urinary dysfunction (6). Meanwhile, active surveillance reduces the harm associated with immediate definitive therapy without a significant impact on clinical outcomes for most individuals with low-risk, as well as some individuals with favorable intermediate-risk, PCa (4, 7, 8) with the small but true risk of disease progression.

Minimally invasive interventions, including focal therapies, have also emerged as a potential treatment option for intermediate-risk PCa (9). The 2022 American Urological Association/American Society for Radiation Oncology guidelines for localized PCa have stated that focal therapies may be considered for patients with intermediate-risk, but not high-risk PCa (4). The goal of focal therapy (FT) is to adequately treat clinically significant PCa (csPCa), defined as ≥Gleason 7 (3 + 4) i.e. Grade Group 2 (GG2) disease, while minimizing the morbidity associated with whole gland treatment (10). Focal therapies selectively target confirmed areas of disease in the prostate, while sparing normal prostatic tissue and surrounding structures, including the delicate neurovascular bundles and urinary sphincter, when possible (11). A targeted approach can help reduce unnecessary treatment of additional tissue, and a smaller target (with a better approach for targeting) can ensure a smaller overall treatment area, with a more rapid recovery (12).

The ability of multiparametric magnetic resonance imaging (MRI) to accurately localize the site of disease in the gland has made focal, organ-sparing therapies a more feasible treatment option (13). While MRI accurately localizes the site of disease in the gland, it underestimates the true extent of the lesion, and therefore, adequate margins of 8-9 mm around the MRI visible tumor have been suggested for successful focal treatment (14, 15). Focal therapies can preserve the quality of life in men with PCa by treating the sites of csPCa, at the most risk for metastasis, while reducing the rate of unwanted effects of therapy, such as erectile dysfunction and stress incontinence (5, 6).

Energy source options for FT include high-intensity focused ultrasound (HIFU), radiofrequency ablation, microwave ablation, cryoablation, irreversible electroporation, and photothermal ablation (2). The use of HIFU in the prostate was first described in 1993, delivered *via* a transrectal approach using ultrasound guidance (16, 17). Since then, US-guided HIFU has been extensively studied (18–21). More recently, the use of MRI-guided HIFU has been assessed for FT with promising results (5, 10).

HIFU utilizes acoustic energy generated by piezoelectric transducers to non-invasively thermally ablate a focal point of targeted tissue, resulting in coagulation necrosis without affecting the intervening and surrounding tissue (1, 22, 23). To achieve this result, temperatures >50°C for 10 seconds or 56°C for 1 second are required; however, in clinical practice, temperatures >65°C for a few seconds are used to achieve tissue necrosis (17, 24). Sonication refers to the deposition of energy into the tissue (25). In targeting a region, multiple sonications are generally performed for successful ablation, with pause intervals between treatments to minimize heat accumulation and destruction of surrounding tissue (1, 25). As with diagnostic ultrasound, the sound waves do not pass through air or dense structures such as bone. Intraprostatic calcifications in the treatment beam path presents a potential limitation to HIFU therapy (2). It reflects ultrasound waves altering sonification of the target tissue (2). This can result in suboptimal or ineffective treatment of PCa undergoing FT. Additionally, the HIFU energy source delivers energy from an extraprostatic location which then leads to higher loss of ablative energy for tumors located at a distance from the point-source (26).

Ultrasound guidance was first used for HIFU of localized PCa using a transrectal approach, however, MRI guidance has been assessed as an alternative option more recently (10). Although MRI-guided HIFU has certain limitations, including the need for additional expertise, resources, and cost, it also has several advantages (10). MRI guidance can more accurately target the tumor site with adequate margins in all 3 planes, allowing for smaller ablation volumes, fewer adverse effects, and a more rapid recovery (22, 27), compared to targeting for FT under ultrasound guidance. Furthermore, MRI-guided FT utilizes MR thermometry which allows for thermal feedback and real-time power adjustment to optimize tissue ablation temperatures (10). Following MR-guided HIFU therapy, gadolinium-based contrast media can also be administered for assessment of the nonperfused volume, as a method of assessing the ablated area (10) and treatment coverage.

There are two MRI-guided HIFU devices which have been tested in large clinical trials, a transrectal device (MRgFUS, Insightec Ltd, Haifa, Israel) and a transurethral device (TULSA-PRO, Profound Medical Inc, Toronto, Canada). In this review, we will discuss the techniques and performance of MRI-guided transrectal HIFU (MRgFUS) and transurethral ultrasound ablation (TULSA) ablation for localized PCa and whole gland treatment.

## Transrectal MR guided focused ultrasound surgery (MRgFUS):

### Technique

**Figure 1:**
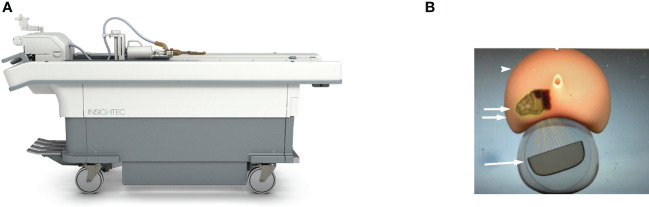
**(A)** Photograph of the ExAblate 2100 Prostate device. **(B)** Graphic image of the ExAblate device (arrow) surrounded by balloon filled with degassed water and steered to the direction of the tumor for treatment. Rectangular boxes (double arrow) within the prostate gland (arrowhead) represent sonication spots within a macrosonication. (Courtesy of InSightec Ltd).

The ExAblate 2100 Prostate device (**Figure 1**) is a transrectal MRgFUS system which delivers HIFU energy into the prostate *via* a minimally invasive approach using an endorectal ultrasound transducer (28, 29). The configuration of the phased-array transducer, which is made of approximately 1000 elements and operates at a frequency of 2.3 MHz and power of 30 W, allows the system to steer the US beam to the desired target within the prostate based on MRI guidance (28). T2-weighted and diffusion weighted images are obtained initially for treatment planning. Thereafter, the prostate gland, tumor site, urethra, rectal wall, and neurovascular bundle are manually contoured. Margins of up to 10 mm beyond the MRI visible tumor, when possible, are included in treatment planning. A Foley catheter is placed at start of procedure for continuous bladder drainage. Occasionally, a suprapubic catheter may have to be placed if the urethra is included in the treatment plan. The endorectal probe is filled with degassed water at 14°C for rectal cooling and protection, as well as to eliminate air within the beam path (30). At the start of treatment, subtherapeutic sonications are administered to confirm the target region. Subsequently, multiple treatment sonications are consecutively delivered to the target area. For achievement of homogenous coagulation, each sonication is overlapped with the previous sonication.

**Figure 2:**
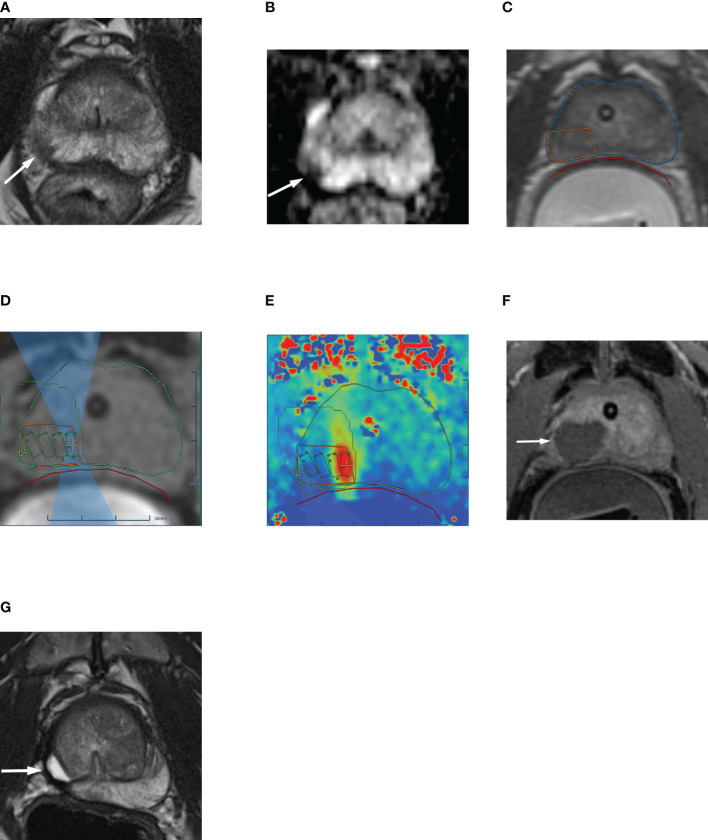
63-year-old man with biopsy confirmed Gleason 7 (3 + 4) prostate cancer undergoing MRgFUS. **(A)** Pre-treatment axial T2-weighted fast spin-echo MR image and **(B)** ADC map image, shows the tumor in right posterolateral peripheral zone (arrow). **(C)** Intra-operative MR image shows the contoured rectal wall (red outline), prostate margin (blue outline) and the region of interest (orange outline). **(D)** Intra-operative MR image shows focused ultrasound beam path (blue) overlaid on treatment plan. The rectangular boxes illustrate each sonication spot. **(E)** Thermal map image obtained during treatment which depicts the temperature with heat deposition color coded in red overlaid on sonication spot. **(F)** Axial gadopentetate dimeglumine-enhanced MR image obtained immediate post treatment shows the devascularized ablated volume (arrow). **(G)** Corresponding T2-weighted fast spin-echo MR image at 24 months post ablation showing involution and volume loss in peripheral zone. Targeted biopsy from treatment zone and rest of gland were negative.

A 10-mm margin beyond the MRI visible tumor volume should be included in the treatment plan where possible, though care should be taken to protect the sensitive structures, such as the rectal wall, external urethral sphincter, bladder wall. If necessary, the urethra can be included within the treatment area, as the prostatic urethra grows new epithelial lining over time (30). During ablation, MR thermography provides real-time temperature feedback of the treated region (29). Dynamic contrast-enhanced MRI is performed following ablation to confirm treatment coverage and the devascularized nonviable treatment area (28). Prostate MRI before, during, and after MRgFUS therapy for a patient with Gleason 7 (3 + 4) PCa are shown in **Figure 2**.

### MRgFUS: Clinical Performance

**Table 1** provides a summary of the characteristics of the major MRgFUS and MR-guided TULSA trials. **Table 2** provides a summary of the key oncologic and functional outcomes of the major MRgFUS and TULSA ablation studies for treatment-naïve PCa.

**Table 1:** Characteristics of prostate MRgFUS and TULSA ablation studies.

First Author	Year	Journal	Treatment Method	Target Disease	Sample Size	Gleason Grade Group	Treatment Target	Study Design
Chopra R	2012	Radiology	TULSA	PCa	8	≤3	Angular sector	Single center
Napoli A	2013	European Urology	MRgFUS	PCa	5	≤3	Focal	Single center
Chin JL*	2016	European Urology	TULSA	PCa	30	≤2	Whole gland	Multi-center
Ramsay E	2017	Journal of Urology	TULSA	PCa	5	≤3	Angular sector	Single center
Tay KJ	2017	Radiology	MRgFUS	PCa	14	1	Focal	Single center
Ghai S	2018	European Radiology	MRgFUS	PCa	8	≤3	Focal	Single center
Anttinen M	2020	Scandinavian Journal of Urology	TULSA	PCa	6	≤4	Focal	Single center
Anttinen M	2020	European Urology Open Science	TULSA	Radiorecurrent PCa	11	≤5	Whole and partial gland	Single center
Klotz L	2021	Journal of Urology	TULSA	PCa	115	≤2	Whole gland	Single center
Ghai S	2021	Radiology	MRgFUS	PCa	44	≤3	Focal	Single center
Nair SM*	2021	British Journal of Urology International	TULSA	PCa	22	≤2	Whole gland	Multi-center
Elterman D*	2021	Journal of Endourology	TULSA	BPH	9	N/A	Whole gland	Multi-center
Ehdaie B	2022	Lancet Oncology	MRgFUS	PCa	101	≤3	Focal	Multi-center
Makela P	2022	Acta Radiologica	TULSA	Radiorecurrent PCa	8	≤5	Whole and hemi-gland	Single center
Viitala A	2022	British Journal of Urology International	TULSA	BPH	10	N/A	Transition zone	Single center

MRI, magnetic resonance imaging; MRgFUS, MRI-guided transrectal high-intensity focused ultrasound; TULSA, transurethral ultrasound ablation; PCa, prostate cancer; BPH, benign prostatic hyperplasia; N/A, not applicable.

*Studies performed with overlapping sample population.

**Table 2:** Key findings of major treatment-naïve prostate cancer MRgFUS and TULSA ablation studies.

First Author	Year	Treatment Method	Sample Size	Treatment Target	Gleason Grade Group (baseline)	Follow-up Period	Oncologic Outcome	Functional Outcome
Chopra R	2012	TULSA	8	Angular sector	GG1: 2 GG2: 4 GG3: 2	N/A	Mean targeting accuracy was -1.0 mm ± 2.6 mm, suggesting tendency for undertreatment. Continuous region of thermal coagulation on histology without prostate capsule inclusion. At prostatectomy, no evidence of thermal effects on surrounding structures.	N/A.
Napoli A	2013	MRgFUS	5	Focal	GG1: 3 GG2: 2	N/A	Extensive coagulative necrosis without residual viable tumor in ablation area on whole-mount prostate section specimens.	N/A.
Chin JL*	2016	TULSA	30	Whole gland	GG1: 24 GG2: 6	12 months	At 12 months, 9/29 residual clinically significant disease (GG1 >10mm, GG2 >3 mm, or any GG3 disease); 16/29 with any residual disease.	Quality of life outcomes returned to pretreatment baseline at 3 months. Erectile function decreased initially, but it returned to pretreatment baseline at 12 months.
Ramsay E	2017	TULSA	5	Angular sector	≤GG3	N/A	Mean spatial target accuracy was -1.5 mm ± 2.8 mm, suggesting tendency for undertreatment. Study demonstrated successful thermal coagulation of angular sectors with inclusion of the capsule.	N/A.
Tay KJ	2017	MRgFUS	14	Focal	GG1: 14	24 months	At 24 months, 8/14 with residual disease (1 GG2 and 1 GG4): 2/8 in-field disease only, 4/8 out-of-field disease; 2/8 with in-field and out-of-field disease.	Urinary symptoms and sexual function normalized at 3 months; no significant functional decline by 24 months.
Ghai S	2018	MRgFUS	8	Focal	GG1: 4 GG2: 2 GG3: 2	6 months	At 6 months, 4/8 with in-field residual PCa: 3/4 with low-volume GG1 and 1/4 with GG4.	Residual urinary/erectile dysfunction in 1/8 patients.
Anttinen M	2020	TULSA	6	Focal	GG1: 1 GG2: 2 GG3: 2 GG4: 1	3 weeks	Target ablations were successful in all patients, with target treatment volumes of 7-19 mL. At prostatectomy, histopathology demonstrated no viable malignancy within the ablated targets.	No difference in quality of life outcomes between baseline and 3 weeks post-TULSA. Normal continence at 3 weeks in all patients.
Klotz L	2021	TULSA	115	Whole gland	GG1: 43 GG2: 69 GG3: 3	12 months	At 12 months, 39/111 with residual disease (22 GG1, 11 GG2, 3 GG3, 2 GG4, and 1 GG5 disease).	Erections were maintained/regained in 69/92 of potent men at 12 months.
Ghai S	2021	MRgFUS	44	Focal	GG2: 36 GG3: 8	5 months	At 5 months, 3/44 with in-field residual disease (≥6 mm GG1 disease or any volume ≥GG2 disease) and 1/44 with out-of-field disease (2mm GG1 disease).	Erectile and urinary function scores similar at baseline and 5 months.
Nair SM*	2021	TULSA	22	Whole gland	≤GG2	3 years	At 3 years, 10/29 residual clinically significant disease (GG1 >10mm, GG2 >3 mm, or any GG3 disease).	Erectile function stable at 3 years.
Ehdaie B	2022	MRgFUS	101	Focal	GG2: 79 GG3: 23	24 months	At 24 months, 11/89 with in-field residual disease (≥GG2 disease; 3/11 with ≥GG4); 39/98 with out-of-field residual disease (≥GG2 disease).	At 24 months, no reported urinary incontinence requiring pad use; slight decrease in sexual function reported.

MRI, magnetic resonance imaging; MRgFUS: MRI-guided transrectal high-intensity focused ultrasound; TULSA, transurethral ultrasound ablation; PCa, prostate cancer; N/A, not applicable.

*Studies performed with overlapping sample population.

One of the earliest experiences of MR-guided HIFU in humans was a proof-of-principle study reported by Napoli et al. (31). Five consecutively enrolled men with unifocal, biopsy proven PCa (Gleason score ≤7; ≤ cT2aN0M0; 3 participants with GG1 disease and 2 with GG2 disease) visible on multiparametric MRI (mpMRI) were treated with the ExAblate device and were then taken to open radical prostatectomy within 2 weeks following the procedure. In all cases, whole-mount prostate section specimens showed extensive coagulative necrosis without residual viable tumor in the ablation area (31).

A Phase I feasibility and safety study by Tay et al. prospectively assessed 14 patients with low volume (≤10 mm^3^), low-grade (GG1; ≤cT2aN0M0) PCa treated with MRgFUS (32). Five patients experienced periprocedural hematuria or mild lower urinary tract symptoms, and 2 experienced urinary tract infections treated with antibiotics. Urinary symptoms and sexual function normalized at 3 months following HIFU, with no other significant functional decline by 24 months. PSA levels decreased by a median of 39% within 3 months following treatment, remaining low in all but one patient. No individual demonstrated positive mpMRI findings at the 6-month or 24-month follow-up. At 6 months, 6 patients had PCa (one of whom had GG2 disease) on transperineal mapping template biopsy (24 cores per patient), one of whom had both in-field and out-of-field disease, while the remaining 5 patients had only out-of-field disease. At 24 months, 8 patients had PCa on template biopsy, one of whom had GG2 disease and another who had GG4 disease. This consisted of 2 individuals with in-field only disease, 4 individuals with out-of-field only disease, and 2 individuals with both in-field and out-of-field disease (32).

Ghai et al. performed a feasibility and safety Phase I prospective study in 8 patients (10 lesions) with low-intermediate risk PCa (≤ cT2a, and Gleason ≤7) treated with the ExAblate device, for which earlier results were published on 4 patients (28, 29). mpMRI was performed in each patient followed by 16-core extended systematic confirmatory biopsy as well as 2-4 additional targeted samples for MRI-visible lesions when present. Six treatment sites (4 participants) had GG1 disease, 2 sites in 2 participants had GG2 disease, and the remaining 2 sites in 2 patients had GG3 disease at baseline. In 6 out of 8 patients, quality of life parameters were similar between baseline and 6 months. At 6 months, mpMRI was negative in all treated patients. On the 6-month biopsy, 3 patients had low volume MR-invisible GG1 residual disease, and one patient had GG4 residual disease at the treatment site. There were no significant periprocedural complications, and urinary and erectile dysfunction rates were low (1/8 patients affected) (29).

A Phase II prospective, single center trial assessing 44 intermediate-risk PCa patients treated with the ExAblate device was conducted by Ghai et al. and the early results were published in 2021 (10). For enrollment each patient required 12-core systematic biopsies in addition to targeted samples of MRI-suspicious sites using fusion MRI-TRUS biopsy. At baseline, 36 patients had GG2 disease, and the remaining 8 patients had GG3 (Gleason 4 + 3) disease. No significant treatment-related adverse events occurred. The median PSA level dropped from 6.4 ng/mL at baseline to 2.4 ng/mL at 5 months after treatment. At 5 months after ablation, 3/44 participants had residual disease (≥6 mm GG1 disease or any volume ≥GG2 disease) at the treatment site on targeted MRI-TRUS fusion biopsy while 41/44 participants (93%) were free of csPCa. Furthermore, new Prostate Imaging Reporting and Data System (PI-RADS) category 3 lesions in the nontreated areas of the prostate were seen in 3/44 individuals on mpMRI at 5 months. None of the patients had out-of-field csPCa (two of the sites were negative for any disease, and one demonstrated 2 mm of GG1 disease at targeted biopsy). Overall, erectile and urinary function scores were similar at baseline and 5 months (10).

Similarly, Ehdaie et al. conducted a Phase IIB, prospective, multi-center trial assessing 101 patients with intermediate-risk PCa (≤ cT2; 79 GG2 & 22 GG3 disease) treated with the ExAblate device (5). Participants underwent either transperineal or transrectal MRI-targeted biopsy including systematic samples at baseline and follow up. At least 2 targeted cores were directed at MRI-visible index lesions. No significant treatment-related adverse events were reported. At 6 months, 96/101 individuals had no evidence of GG2 or higher disease in the targeted treatment area and 77/101 individuals had no evidence of GG2 or higher disease anywhere in the prostate gland on MRI-targeted and systematic biopsy. At 24 months, 78/89 individuals had no evidence of GG2 or higher disease in the targeted treatment area and 59/98 individuals had no evidence of GG2 or higher disease anywhere in the prostate gland on MRI-targeted and systematic biopsy. Of the 11 individuals with residual disease detected in the treatment area at 24 months, 3 had GG4 (Gleason 8) or higher disease. Serum PSA levels decreased following treatment and stabilized at 6 months, after which a slight increase was seen at 24 months which may be related to increase in gland size over time. The mean decrease in serum PSA following treatment was 3.0 ng/mL at 6 months and 2.6 ng/mL at 24 months. By 24 months, no patient reported urinary incontinence requiring pad use, while a slight decrease in sexual function was found (5).

These recent two trials (5, 10) have shown that MRgFUS FT is safe and has good oncologic and results at the treatment site and reasonable quality of life outcomes (5, 10). Notably, these trials screened all recruited patients with CT, and individuals with significant intraprostatic calcifications close to the rectum or within the treatment beam path were excluded. This may have contributed to better oncological outcomes compared to other published results for HIFU FT, as intraprostatic calcifications can limit the efficacy of HIFU. MRgFUS may also be limited in anterior gland target lesions, due to the anatomical limitation of a transrectal approach (26). Additionally, in the study by Ehdaie et al, a significant number of patients had csPCa outside of the treatment area (39/98 at 2 years) (5). This may to some extent be related to the changing clinical practise of MRI targeted biopsy only and omission of systematic biopsies. A recent study found avoiding systematic biopsies may only be judicious in patients with prior negative biopsy or in those who had large PIRADS 5 disease and would anyways not be eligible candidates for FT (33). Furthermore, initial studies have shown higher diagnostic performance of prostate-specific membrane antigen (PSMA) positron emission tomography (PET)/MRI compared to MRI alone and may therefore help in better patient selection for FT in the future (5, 12).

Multiple prior studies have assessed the oncological outcomes of US-guided HIFU FT for localized PCa. For example, Guillaumier et al. performed a multi-center trial of 625 men with csPCa (166 GG1, 327 GG2, 86 GG3, and 11 ≥GG4 disease; 9 not reported) treated with focal US-guided HIFU (18). A total of 222 patients underwent biopsy following focal HIFU, of which 40 (18%) had in-field recurrence, and 27 (12%) had out-of-field recurrence (of these, 11 had both in- and out-of-field recurrence) (18). A prospective, single-arm study of 42 individuals with low- and intermediate-risk PCa (13 GG1, 24 GG2, and 4 GG3 disease) undergoing focal US-guided HIFU was performed by Ahmed et al. (34). At 6 month follow-up, 9/39 (23%) patients who underwent biopsy demonstrated in-field residual PCa (6 with GG1 and 3 with GG2 disease). (34). A prospective, single center study by Ahmed et al. assessed 56 individuals with PCa (7 low risk, 47 intermediate risk and 2 high risk) undergoing focal US-guided HIFU (11). At 6 month biopsy, 42/52 (81%) individuals had no evidence of csPCa (11). A prospective, single-institution trial of 72 individuals with low- and intermediate-risk PCa undergoing hemi-gland US-guided HIFU ablation was performed by Feijoo et al. (20) however, a majority of the included patients 58/67, 87%, were treated for GG1 disease (20). In comparison with US-guided HIFU, MRgFUS has demonstrated an overall higher rate of success in the targeted treatment of focal PCa despite a rigorous protocol and 2-year follow up (5). This may be in part due to the improved tumor targeting of MRI, as well as the real-time monitoring of the treatment area with MR-thermography. In terms of treatment time, Ghai et al. reported a median “magnet time” (MRI to recovery room) of 256 minutes, and a median ablation time of 125 minutes for MRgFUS (10). Meanwhile, complete procedure times for US-guided HIFU are lower, with studies reporting periods <120 minutes (11, 20, 34).

### MR-Guided TULSA: Technique

**Figure 3:**
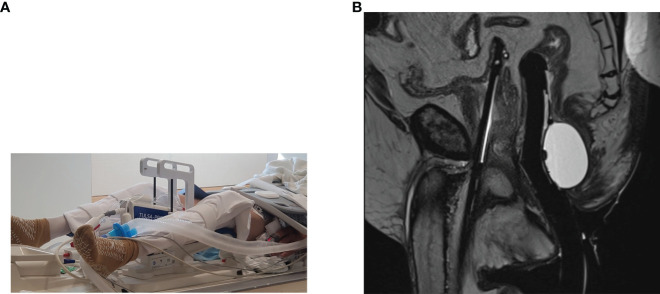
**(A)** Photograph of the TULSA-PRO device. **(B)** T2-weighted sagittal intraprocedural MRI demonstrating the TULSA-PRO device *in situ* within the urethra, as well as an intra-rectal balloon for cooling. (Images provided by Dr Sandeep Arora, Yale School of Medicine).

**Figure 4:**
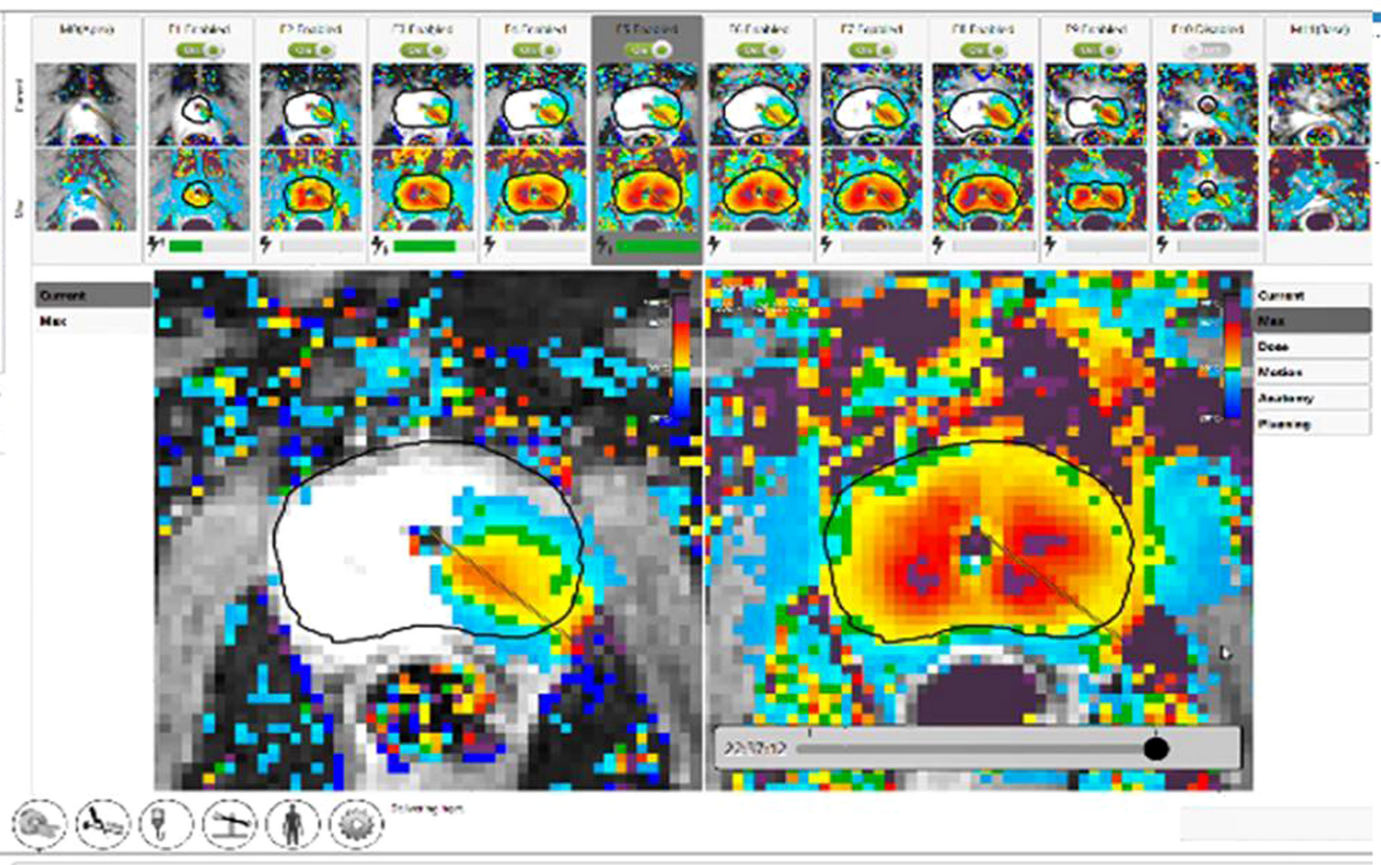
Completion of TULSA procedure. Clockwise ablation of the whole gland with real time dose on the left and cumulative dose on the right. The thermal map demonstrates gradient temperatures. (Image provided by Dr Steven Raman, University of California, Los Angeles).

MRI-guided TULSA was developed in 2012 as a potential new means of MR-guided FT for PCa (2). **Figure 3** demonstrates the TULSA-PRO device (A), as well as an intra-procedural MRI of the TULSA-PRO device *in situ* in a patient undergoing MRI-guided TULSA (B). TULSA utilizes a transurethral approach to deliver prostate ablative therapy, rather than a transrectal one (35). Although initial TULSA studies tested whole-gland ablation, partial gland and focal/segmental therapies have been performed more recently (2). Similar to MRgFUS, TULSA takes advantage of MRI for PCa localization, as well as MR thermometry to monitor and guide prostate tissue ablation (35). The TULSA-PRO device provides rectal and urethral cooling during treatment (2, 35). For the TULSA-PRO device, a rigid ultrasound applicator incorporates a linear array of 10 independent ultrasound transducers (36). These transducers emit high-intensity US energy in a directional, rather than focused, manner directly into the adjacent prostate. This configuration allows US beams to interact with a large volume of tissue, resulting in shorter treatment times and more consistent thermal ablation areas (36). During the procedure, the ultrasound applicator is positioned within the prostatic urethra, with a safety margin of 3 mm between the transducers and the sphincter at the prostate apex (36). The target prostate volume is heated to ≥55°C to achieve acute thermal coagulation under MRI-guidance. High-intensity ultrasound energy is delivered to the prostate target area *via* rotation of the ultrasound applicator with simultaneous MRI thermometry feedback control (**Figure 4**). Maximum prostate temperatures are maintained <100°C to avoid tissue carbonization and boiling. Following treatment, contrast-enhanced MRI is acquired to assess the non-perfused volume (36). Prior to commencement of the procedure, a suprapubic catheter is placed, for continuous bladder drainage and to avoid prostate movement between treatment planning and real-time MRI thermometry. The suprapubic catheter is generally left in place for up to 2 weeks following TULSA to avoid acute urinary retention secondary to thermally induced edema (36).

### MR-guided TULSA: Clinical performance

A prospective pilot feasibility and safety study (proof-of-principle) performed by Chopra et al. assessed the use of focal MR-guided TULSA in 8 individuals with Gleason ≤7 PCa (≤ cT2a disease, 2 men with GG1 disease, 4 with GG2 disease and remaining 2 individuals with GG3 disease) prior to radical prostatectomy (35). Focal 180° angular sector volumes within the posterior region of the prostate gland were targeted for treatment, with a target boundary placed at a safety margin of at least 6 mm from the prostate capsule. No significant periprocedural events occurred, and the treatment was well tolerated by all patients. To compare findings at MRI to whole-mount histologic examination, spatial targeting accuracy was assessed, defined as the radial distance between the 55°C isotherm and the target boundary, and measured at every degree along the target boundary. The mean targeting accuracy was -1.0 mm ± 2.6 mm, which suggested a tendency for undertreatment. Histologic examination revealed a continuous region of thermal coagulation. Excellent spatial correspondence was seen between the spatial heating pattern and pattern of thermal damage in gland. Furthermore, at prostatectomy, the surgeon reported normal structure and consistency of the exterior of the prostate gland, and no evidence of thermal effects on surrounding structures.

While multiple studies with the TULSA-PRO device have assessed subtotal gland ablation, there have been few recent studies assessing outcomes following sectoral FT. Ramsay et al. performed a prospective safety and feasibility study in 5 individuals with Gleason ≤7 PCa (≤ cT2a disease) (9). Each individual underwent focal MR-guided TULSA treatment of angular sectors with inclusion of the capsule, followed by radical prostatectomy. Comparison of MRI to whole-mount histology demonstrated a mean spatial target accuracy was -1.5 mm ± 2.8 mm, based on the radial distance between the 55°C isotherm and the target boundary, again suggesting a slight tendency for undertreatment. Overall, the trial found that MR-guided TULSA can thermally coagulate angular sectors with inclusion of the capsule in prostate gland volumes up to 70 cc (9).

Anttinen et al. performed a prospective, single-center, Phase I trial assessing lesion-targeted FT TULSA followed by robot-assisted laparoscopic prostatectomy after 3 weeks in 6 participants (8 lesions) with MRI-visible, biopsy-confirmed histologically significant (≥GG2 disease or GG1 >6 mm, >50% in any core, or >2 positive biopsy cores) localized PCa (37, 38). Of the 6 patients, 1 had GG1, 2 had GG2, 2 had GG3, and 1 had GG4 disease. Target ablations were successful in all patients, with target treatment volumes of 7-19 mL and treatment radii measuring up to 33.6 mm. No significant treatment-related complications occurred. No difference in quality of life outcomes between baseline and 3 weeks post-TULSA, and all patients had normal continence at 3 weeks. Non-perfused volumes determined with MRI covered the ablation targets and increased 36% at 3 weeks, which correlated with necrotic areas at histology. Mean histological demarcation between the inner border demonstrating complete necrosis and the outer border demonstrating thermal injury was 1.7 ± 0.4 mm. Coagulation necrosis extended to the level of the capsule, except at the neurovascular bundles, where a safety margin of 3 mm was used. At prostatectomy, histopathology demonstrated no viable malignancy within the ablated targets (37, 38).

A prospective, multi-center Phase I safety and feasibility trial by Chin et al. across 3 tertiary referral centers assessed whole gland MR-guided TULSA ablation (with the intent of preserving 10% residual viable prostate gland at capsule) in 30 individuals with low- and intermediate-risk PCa (≤ cT2a disease; 24 with GG1 and 6 with GG2 disease) (36). There were no major periprocedural adverse events or rectal injuries; adverse events included hematuria (43%), urinary tract infections (33%), acute urinary retention (10%), and epididymitis (3%). Mean thermal ablation accuracy and precision, referring to mean and standard deviation of the spatial distance between the 55°C isotherm to the target prostate boundary during treatment and the target prostate boundary defined during treatment planning, were 0.1 mm ± 0.4 mm and 1.3 mm ± 0.4 mm, respectively. Quality of life outcomes returned to pretreatment baseline at 3 months, and symptoms improved compared to baseline in 17 patients. Erectile function decreased initially, but it returned to pretreatment baseline at 12 months based on median International Index of Erectile Function scores. Of 20 individuals with erections sufficient for penetration at baseline, 17 remained at 12 months. Median PSA decreased 87% from baseline (5.8 ng/mL) to 1 month (0.8 ng/mL) and remained stable at 12 months (0.8 ng/mL). At 12-month follow-up, 9/29 (31%) patients had a positive 12-core transrectal, US-guided biopsy for csPCa (GG1 >10mm, GG2 >3 mm, or any GG3 disease), while 16/29 patients had a positive biopsy for any disease (36). A subsequent study completed 3 years of follow-up in 22 individuals from the original study (39), during which no new serious or severe adverse events were reported. Erectile function and PSA levels were also stable at 3 years. Overall, 10/29 (34%) participants had csPCa at 3 years, including 19 men who had repeat biopsy at the 3-year mark, and 10 men who only completed biopsy at 12 months (39).

**Figure 5:**
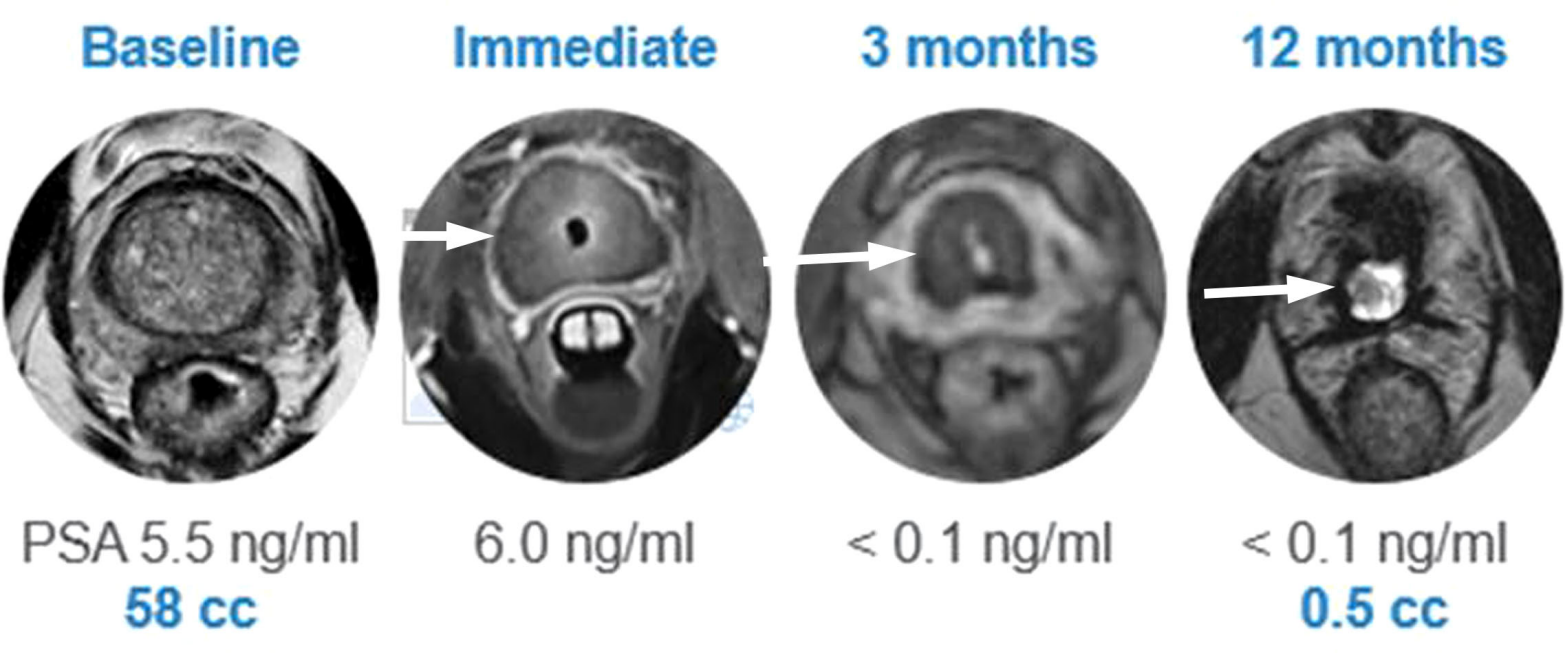
Post TULSA appearance. MRI appearance at baseline, immediate post treatment, at 3 and 12 months after ablation showing progressive reduction in prostate volume (arrow). (Image provided by Dr Steven Raman, University of California, Los Angeles).

A recent multi-center prospective trial by Klotz et al. at 13 sites included 115 men with low- to intermediate-risk PCa (≤ cT2b disease; 43 with GG1, 69 with GG2, and 3 with GG3 disease) undergoing MRI-guided whole gland TULSA (to the prostate capsule) (40). Of note, 14/115 individuals were reported to have intraprostatic calcifications <10 mm at screening, however, the precise method of screening was not reported. Periprocedural adverse events included genitourinary infection (4%), urethral stricture (2%), urinary retention (2%), urethral calculus with pain (1%), and urinoma (1%) which all resolved at the 12-month follow-up. No severe adverse events or rectal injuries were reported. Erections were maintained/regained in 69/92 (75%) of potent men at 12 months. The primary endpoint of the study, a PSA reduction of ≥75%, was achieved in 110/115 (96%) of patients. Median PSA decreased from 6.3 ng/mL at baseline to 0.5 ng/mL at 1 month, and remained stable at 12 months, measuring 0.5 ng/mL. The median prostate volume was reduced from 37 cc to 3 cc (**Figure 5**). At 12 months, 72/111 (65%) of individuals who underwent biopsy demonstrated no evidence of any PCa. Of the remaining 39/111 with biopsy-detected PCa at 12 months, 22 had GG1, 11 had GG2, 3 had GG3, 2 had GG4, and 1 had GG5 (Gleason score 9-10) disease. Predictors of persistent disease included intraprostatic calcifications, suboptimal MRI thermal coverage of the targeted area, and positive mpMRI findings at 12 months (40).

Anttinen et al. performed a prospective, phase I, single-arm, single-center trial assessed the role of salvage TULSA in 11 individuals with localized, histopathologically verified, radiorecurrent PCa (7 with GG1, 1 with GG2, 1 with GG3, and 2 with GG5 disease) (41). Whole-gland TULSA was performed in 3 patients, while partial gland TULSA was performed in 8 patients. Of note, 5/11 patients had fiducial seeds in situ. Patients were followed for 12 months, and underwent PSMA PET/CT, mpMRI, and TRUS-guided biopsy at that point. Salvage TULSA was feasible in each patient, with reported post-treatment adverse events of urinary retention and/or infection in 4 patients. Patients reported a modest decline in functional status, including a decrease in maximum flow rate of 24%. Median PSA decreased from 7.6 ng/mL at baseline to 0.23 ng/mL at 12 months (97% decrease), while median prostate volume decreased by 55% at 12 months. At 12 months, 1 in-field (partial gland TULSA) and 2 out-of-field (1 partial gland and 1 whole-gland TULSA) histopathologically confirmed recurrences occurred. One patient with in-field recurrence following partial gland TULSA successfully underwent repeat salvage TULSA (41).

Makela et al. performed a retrospective, single center, phase I trial assessing salvage TULSA in 8 patients (2 men with GG1, 2 with GG2, 1 with GG3 and 3 with GG5 disease) with fiducial markers (18 intraprostatic gold seed fiducial markers) to a control cohort of 13 patients who underwent salvage TULSA without fiducial markers (42). Adverse events and functional outcomes were comparable between the 2 cohorts. At 12 months, 11/18 fiducial markers disappeared *via* tissue sloughing. The authors concluded that salvage TULSA for radiorecurrent PCa in the presence of intraprostatic fiducial gold markers was safe and feasible (42).

The use of MRI-guided TULSA has also been assessed for benign prostatic hyperplasia (BPH) with lower urinary tract symptoms (43) A prospective, single-institution, single-arm Phase I safety and feasibility trial enrolled 10 individuals with refractory symptomatic BPH who were previously scheduled to undergo transurethral resection of prostate (TURP). The ablation was planned to cover the adenomatous tissue in the transition zones, with as much extension into the bladder neck as possible. Post-treatment, a total of 4 adverse events (epididymal abscess, prolonged catheterization and UTI, urinary retention) in 3 patients were noted, which resolved in 3 months. At 12 months, no urethral strictures were noted on cystoscopy, and median increases in average flow rate and voided volume were 67% and 20%, respectively. Pre-treatment prostate volumes ranged from 31 mL to 81 mL, and median planned ablation volume was 31 mL. Median prostatic volume decreases at 12 months were 32.5 mL (33% reduction). Median PSA decreased from 3.4 ng/mL at baseline to 1.8 ng/mL at 12 months (48% reduction) (43).

A subset of 9 patients from the prospective, multi-center study by Chin et al. (36) who underwent TULSA to ablate 90% of the prostate gland for localized PCa with lower urinary tract symptoms associated with BPH were retrospectively assessed (44). At 12 months post-TULSA, there was an overall improvement in urinary symptom relief, quality of life, and urinary continence. International Prostate Symptom Score (IPSS) scores significantly improved from 16.1 ± 3.8 at baseline to 6.3 ± 5.0 at 12 months, and IPSS quality of life also improved from 2.8 ± 1.1 to 0.8 ± 1.0. Postvoid residual significantly improved from 95.0 ± 117.5 mL at baseline to 62.5 ± 88.2m mL at 12 months. Perfused prostate volume, measured on MRI, demonstrated a 70% decrease to 13.6 mL at 12 months (44).

Similar to MRgFUS, MRI-guided TULSA has demonstrated promising initial results as a safe and effective treatment option in select low-intermediate risk PCa patients for both FT and whole-gland ablation (36, 39, 40), and multiple registered trials are currently recruiting or in progress assessing the efficacy of TULSA for PCa and BPH (45). Acceptable oncologic profiles and reasonable quality of life outcomes have been reported thus far (36, 39, 40). Mean procedure times of TULSA of 161 minutes (range 104-218 minutes) have been reported (35). Of note, the use of CT for screening of prostate calcifications was not clearly stated in the major TULSA trials, and this may have resulted in an increased number of patients with significant intraprostatic calcifications affecting treatment efficacy. Furthermore, while monitoring of PSA levels plays a vital role following whole-gland treatment, MRI outperforms PSA in the detection of residual or recurrent disease following FT (46, 47). These factors may play an important role in the study design of subsequent TULSA trials for focal prostate therapy.

## Conclusion

MRgFUS and TULSA have shown reasonable oncological and functional results in the treatment of low- to intermediate-risk PCa. Recent Phase II MRgFUS trials have shown better oncologic outcomes than the published results for focal ultrasound guided HIFU and may therefore justify the additional costs associated with MRI guidance. While initial studies on TULSA have focused on subtotal gland ablation, recent trials assessing oncological outcomes following angular sector therapy have shown promise. Overall, the recent major trials for MRgFUS and TULSA support the use of these techniques for focal therapy in appropriately selected patients with low-intermediate risk PCa patients, and ongoing studies are warranted to support their implementation. In addition, TULSA may have a larger role to play in management of BPH symptoms.

## Author contributions

All authors contributed to the article and approved the submitted version.
